# Plasma oxylipin profiling reveals the step-wise activation of ARA/5-HETE metabolism in diabetic kidney disease

**DOI:** 10.1016/j.jlr.2025.100918

**Published:** 2025-10-01

**Authors:** Chunyu Zhou, Xianhui Liang, Jiao Wang, Jia Guo, Qing Zhang, Pei Wang

**Affiliations:** 1 Blood Purification Center, The First Affiliated Hospital of Zhengzhou University, Zhengzhou, Henan Province, China; 2 Department of Geriatric Endocrinology, The First Affiliated Hospital of Zhengzhou University, Zhengzhou, Henan Province, China; 3 Henan Province Research Center for Kidney Disease, Zhengzhou, Henan Province, China; 4 Nephrology Research Center, The First Affiliated Hospital of Zhengzhou University, Zhengzhou, Henan Province, China

**Keywords:** oxylipin, arachidonic acid, 5-HETE, metabolomics, diabetic kidney disease

## Abstract

Diabetic kidney disease (DKD) is a highly prevalent complication of diabetes concomitant with disordered oxylipin metabolism. Our study characterizes the plasma oxylipins associated with the step-wise progression of DKD. An ultraperformance LC-MS/MS method was performed to quantify 141 kinds of oxylipins in plasma samples of patients with DKD or type 2 diabetes mellitus and healthy individuals, both in the test cohort (n = 40 for each group) and the validation cohort (n = 20 for each group). The key oxylipins associated with DKD were identified by orthogonal partial least-squares discriminant analysis and receiver-operating characteristic curve. Polynomial regression, Pearson’s correlation, and logistic regression analyses were performed to assess their correlation with the clinical indicators reflecting DKD progression as well as their diagnostic abilities. Our oxylipin profiling presented the significant alterations of 55 kinds in the test cohort and 42 kinds in the validation cohort. Arachidonic acid (ARA), 5-hydroxyeicosatetraenoic acid (5-HETE), 5-oxoETE, 12-HETE, and 13(S)-HpODE in the test cohort, as well as ARA, 5-HETE, 5-oxoETE, 20-hydroxyPGF2α, and 8,9-EET in the validation cohort, were screened as the key oxylipins distinguishing the DKD group. The increased plasma levels of ARA, 5-HETE, and 5-oxoETE were strongly correlated with estimated glomerular filtration rate. The diagnostic model combining the plasma levels of ARA, 5-HETE, and 5-oxoETE indicated an excellent diagnostic performance for DKD. Collectively, our study disclosed the profiling of oxylipin metabolism, implicating the activation of ARA/5-HETE metabolism associated with the step-wise progression of DKD, which provides the basis for early identification and therapeutic strategies for DKD.

Diabetic kidney disease (DKD) is a highly prevalent complication of diabetes with an incidence reaching epidemic proportions, being estimated to affect more than 180 million of the global population, including a significant number who will develop kidney failure requiring renal replacement therapy (1, 2). Although hyperglycemia acts as an initiating and sustaining factor to continuously damage the kidney, metabolic derangement is also considered the cornerstone of the pathogenesis of DKD due to multiple metabolic adaptations occurring associated with the condition (3). Previous studies have disclosed the abnormal metabolism related to the diseases’ unique metabolic signatures, providing knowledge of the very foundation of several physiological and pathophysiological processes of DKD (4). Our recent studies have successfully related plasma, urine, and salivary metabolites with the onset and progression of DKD, identifying several potential biomarkers among different molecular classes, including amino acids and bile acids (5, 6, 7). The study of metabolic profiling and the discovery of key metabolites have not only illustrated a better understanding of DKD pathogenesis but also brought encouraging perspectives toward its early diagnosis, effective therapeutic approach, or prognosis.

Oxylipins are the epoxide metabolites of PUFAs, derived from the actions of a suite of enzymes, including those in the cyclooxygenase, lipoxygenase (LOX), soluble epoxide hydrolase, and cytochrome P450 families, as well as by nonenzymatic oxidation. Both PUFAs and oxylipins have been well documented to have multiple functions in many physiological, pathophysiological, and pharmacological processes (8, 9). However, controversies exist regarding their pros and cons in kidney diseases. Albeit ω-6 PUFAs are typically considered precursors of proinflammatory and prooxidative oxylipins, their dietary intake has been associated with the reduction of inflammatory state, which is usually present in kidney diseases (10, 11, 12). Meanwhile, studies of the ω-3 PUFAs and their epoxide metabolites presented the limited and preventive roles in IgA nephropathy, acute kidney injury, chronic kidney disease, and end-stage renal disease on dialysis treatment (13, 14). Regarding DKD, numerous studies suggested that ω-3 PUFAs could reduce albuminuria; yet another two clinical studies proposed the opposite conclusion (15, 16, 17). Although oxylipins seem to aggravate DKD progression, insufficient evidence exists to support such an effect. It is still unclear how and to what extent their intervention would benefit kidney disorders.

In this study, we quantified the representative plasma oxylipins using an ultraperformance LC-MS/MS (UPLC-MS/MS)-based metabolomic platform to investigate the alterations of oxylipin metabolism involved in DKD pathogenesis, and the correlation of key oxylipins with clinical indicators reflecting DKD progression. The diagnostic models were also established to assess the diagnostic abilities of the key oxylipins.

## Materials and methods

### Study design and sample collection

This study was approved by the independent Ethics Committee of the First Affiliated Hospital of Zhengzhou University (2020-KY-363) and complied with the World Medical Association Declaration of Helsinki regarding the ethical conduct of research involving human subjects. Patients with DKD (DKD group) and patients with type 2 diabetes mellitus (T2DM group) were recruited from the Department of Nephrology and Department of Endocrinology, respectively. Healthy individuals (CON group) were randomly selected from the Department of Physical Examination to maintain gender and age matched at a statistical significance >0.05 when compared with the patients with DKD or T2DM. The inclusion and exclusion criteria for all participants were as previously described (5). Written and informed consents were obtained from all participants.

The blood samples were collected from all participants in the morning after overnight fasting with EDTA as an anticoagulant. The plasma was separated with centrifugation at 1,500 *g* for 10 min at 4°C within 1 h after collection and then immediately frozen at −80°C until further analysis.

### Chemical and reagents

Oxylipins and the isotope-labeled mix of oxylipins were purchased from Cayman Chemical. HPLC-grade acetonitrile (ACN) and methanol (MeOH) were purchased from Merck (Darmstadt, Germany). MilliQ water (Millipore, Bradford) was used in all experiments. Acetic acid was purchased from Sigma-Aldrich. CNW Poly-Sery MAX solid phase extraction (SPE) cartridges were from ANPEL Co (Shanghai, PRC).

### Preparation of solution and sample for oxylipin detection

The stock solutions of 141 kinds of oxylipins (5 μg/ml) were prepared by dissolving standard compounds in MeOH. Isotope-labeled mix of 25 kinds of oxylipins was diluted with MeOH at a concentration of 100 nM to act as an internal standard (IS). Working solutions were prepared by diluting stock solutions into different gradients (0.01–400 nM) with MeOH.

The plasma samples were prepared using an SPE method. Briefly, 100 μl thawed plasma and 200 μl MeOH/ACN (1:1, v/v) solution containing IS were mixed and vortexed at 2,500 rpm for 10 min and then put at −20°C for 30 min. After centrifugation at 12,000 rpm for 10 min at 4°C, the supernatant was collected and transferred. The collected supernatant was then loaded onto Poly-Sery MAX SPE columns (ANPEL). The eluent was dried under vacuum and redissolved in 100 μl of MeOH/water (1:1, v/v) for further UPLC-MS/MS analysis.

### UPLC-MS/MS-based lipidomic analysis

The details of instrumental analysis were performed according to the protocol reported previously (18). The reconstituted samples were loaded onto a Waters ACQUITY UPLC HSS T3 C18 column (100 mm × 2.1 mm, 1.8-μm particle size; Waters, Milford, MA). The isocratic gradient elution program was run with mobile phase A (ACN/water, 60/40, v/v with 0.002% acetic acid) and mobile phase B (ACN/isopropanol, 50/50, v/v). The gradient program of the LC mobile phase is presented in Supplemental Table S1.

Oxylipins were then detected by AB 6500+ QTRAP LC-MS/MS System (Applied Biosystems Sciex, Toronto, Canada), equipped with an ESI Turbo Ion-Spray Interface, operating in linear ion trap and triple quadrupole scans with negative ion modes and controlled by Analyst 1.6.3 software (Sciex). Oxylipins were analyzed using a scheduled multiple reaction monitoring model with optimized MS conditions (Supplemental Table S2). The acquired chromatograms were analyzed by OriginLab (OriginLab, Northampton, MA). The peaks were identified according to the retention time and specific multiple reaction monitoring transitions of the standards of oxylipins. The concentrations of the oxylipins were calculated against the calibration curve with standards. The mass spectrometer parameters of oxylipins and ISs are listed in Supplemental Table S3 and Supplemental Table S4.

The calculated plasma levels of oxylipins were imported into SIMCA-P v18.0.0.372 (Umetrics Suite; Sartorius, Umeå, Sweden). An orthogonal partial least squares-discriminant analysis (OPLS-DA) model was established to explore the group discrimination. The shared and unique structure (SUS) plot and variable importance in projection (VIP) analyses were generated to determine the major latent oxylipins in the data matrix and contributions of each metabolite to the group discrimination. VIP value >1.2 was considered as statistical significant in terms of discriminating between groups.

### Statistical analysis

Continuous data are presented as mean ± standard deviation. The Shapiro-Wilk test was used to verify the data normality for the comparison between groups. Statistical significance of continuous data was determined using Student’s *t*-test when data were normally distributed; otherwise, significance was determined by the Mann-Whitney *U* test, whereas the categorical data were analyzed by the Chi-square test and Fisher’s exact test, using Prism v8.0.2 software (GraphPad, San Diego, CA). Polynomial regression analyses and Pearson’s correlation coefficients were performed to assess the correlation of key oxylipins with the clinical indicators reflecting DKD progression. *R*^2^ > 0.7 was considered as noticeable correlativity. Logistic regression and the diagnostic models were also performed to evaluate the diagnostic abilities of the key oxylipins. Receiver-operating characteristic (ROC) curves were performed with SPSS, v21.0 (Armonk, NY). The corresponding area under the ROC curve (AUROC), cutoff value, sensitivity, specificity, and accuracy were evaluated to understand the predictive performances of the key oxylipins for DKD. *P* < 0.05 was considered the level of significance.

## Results

### Metabolic profiling of plasma oxylipins

**Fig. 1:**
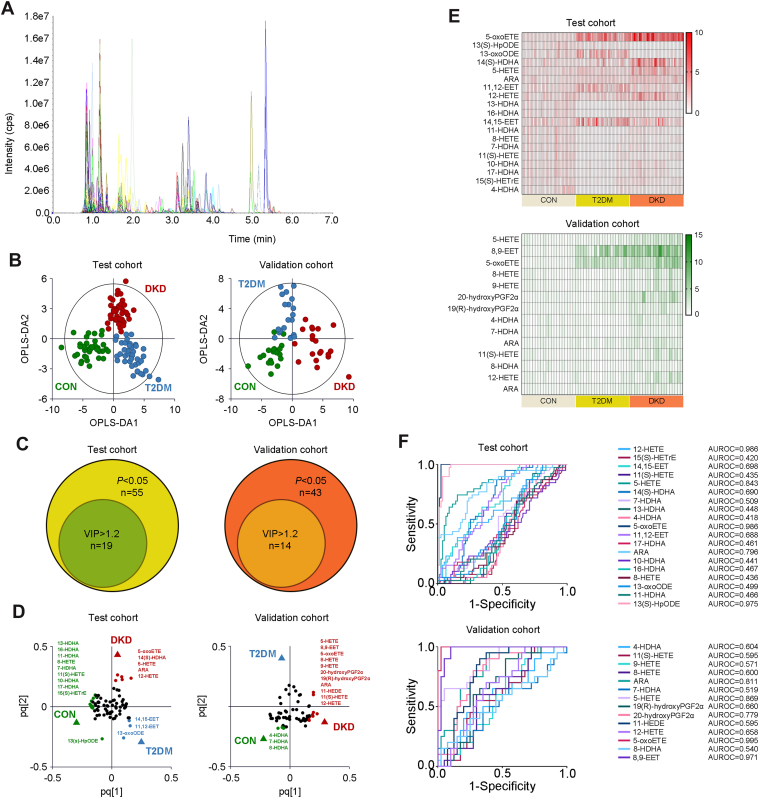
Plasma profiling of oxylipins in the step-wise alterations of DKD. A: Chromatogram of 141 oxylipins in MRM mode. B: The OPLS-DA analyses of the plasmaoxylipin profiling in the first test cohort and the second validation cohort from the DKD, T2DM, and CON groups. C: The Venn diagrams for overlap of key oxylipins with *P* < 0.05 and VIP value >1.2 in the test and validation cohorts. The loading plots (D), heatmap (E), and the ROC curves (F) of the preliminarily selected differential metabolites in the test and validation cohorts. The detailed information relating to the chromatogram of each oxylipin is listed in Supplemental Table S3. MRM, multiple reaction monitoring.

The plasma from 40 individuals in each group was collected and detected as the first test cohort, whereas the plasma from 20 individuals in each group was collected and detected as the second validation cohort, to validate the metabolomic profiling in the test cohort, from January 2023 to December 2023. The demographic characteristics of the study population were extracted from the medical record system of the hospital and are presented in Supplemental Table S5. Of 141 kinds in the established UPLC-MS/MS method (Fig. 1A), oxylipins with >30% of the measurements below the lower limit of quantitation were excluded. As a result, 77 kinds of oxylipins were detected in the test cohort, whereas 65 kinds were detected in the validation cohort. Within the detected oxylipins, 55 kinds in the test cohort and 42 kinds in the validation cohort were significantly altered in the DKD group compared with those in the T2DM group or the CON group (Supplemental Table S6 and Supplemental Table S7).

The OPLS-DA analysis was then performed, highlighting the optimal discrimination among the DKD, T2DM, and CON groups in the test cohort (Fig. 1B; R^2^X = 0.415, R^2^Y = 0.789, and Q^2^ = 0.706) and validation cohort (R^2^X = 0.495, R^2^Y = 0.668, and Q^2^ = 0.572), with optimal binary classifier, validity, and degree of overfitting (Supplemental Fig. S1). According to the significance of *P* < 0.05 in the Student’s *t*-test and VIP >1.2 in the OPLS-DA model, 19 kinds of oxylipins in the test cohort, including 5-oxoETE, 13(S)-HpODE, 13-oxoODE, 14(S)-HDHA, 5-hydroxyeicosatetraenoic acid (5-HETE), ARA, 11,12-EET, 12-HETE, 13-HDHA, 16-HDHA, 14,15-EET, 11-HDHA, 8-HETE, 7-HDHA, 11(S)-HETE, 10-HDHA, 17-HDHA, 15(S)-HETrE, and 4-HDHA, as well as 14 kinds in the validation cohort, including 5-HETE, 8,9-EET, 5-oxoETE, 8-HETE, 9-HETE, 20-hydroxyPGF2α, 19(R)-hydroxyPGF2α, 4-HDHA, 7-HDHA, ARA, 11(S)-HETE, 8-HDHA, 12-HETE, and ARA are the key oxylipins contributing to the group differences between the groups (Fig. 1C–E). Among these oxylipins, 12-HETE, 5-HETE, 5-oxoETE, ARA, and 13(S)-HpODE, in the test cohort, as well as ARA, 5-HETE, 20-hydroxyPGF2α, 5-oxoETE, and 8,9-EETE in the validation cohort, showed the optimal predictive abilities with ROC values >0.7 (Fig. 1F).

### The separated oxylipin profiling in the test cohort

**Fig. 2:**
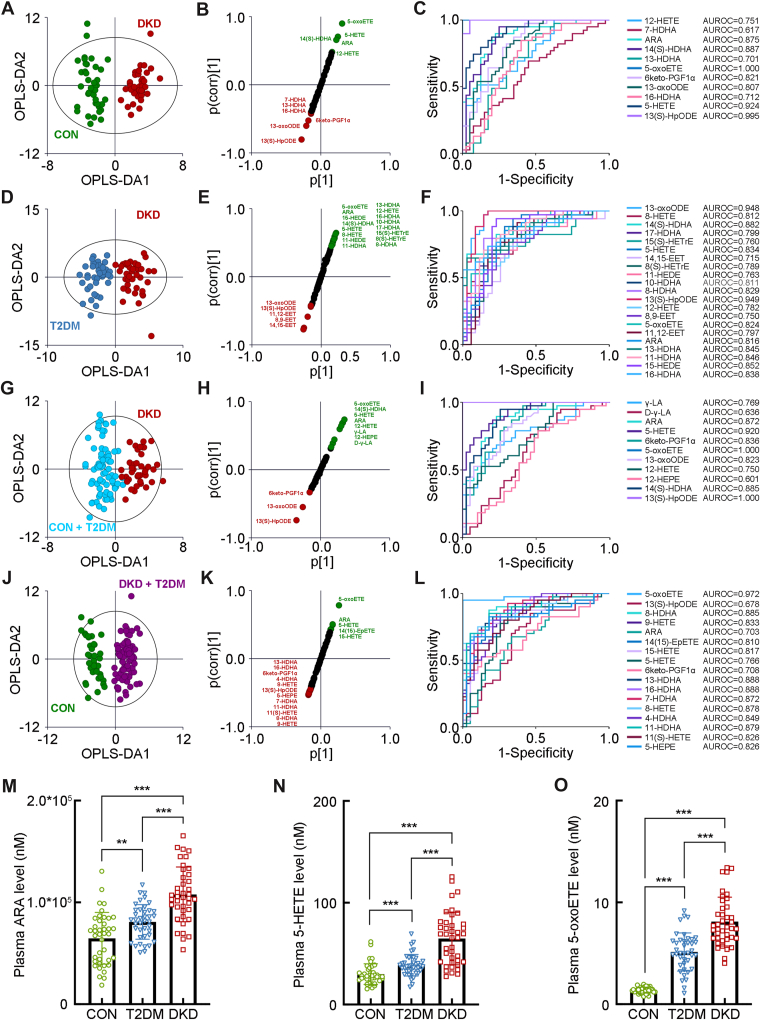
The separated metabolomic profiling in the first test cohort. A: The OPLS-DA score plot between the DKD group and the CON group. The SUS plot (B) and ROC curve analyses (C) of the key oxylipins between the DKD group and the CON group. D: The OPLS-DA score plot between the DKD group and the T2DM group. The SUS plot (E) and ROC curve analyses (F) of the key oxylipins between the DKD group and the T2DM group. G: The OPLS-DA score plot between the DKD group and the non-DKD group (both the TDM group and the CON group). The SUS plot (H) and ROC curve analyses (I) of the key oxylipins between the DKD group and the non-DKD group. J: The OPLS-DA score plot between the group in diabetic condition (both the DKD and the T2DM group) and the CON group. The SUS plot (K) and ROC curve analyses (L) of the key oxylipins between the group in diabetic condition and the CON group. The plasma levels of the key oxylipins, including (M) ARA, (N) 5-HETE, and (O) 5-oxoETE. ∗∗*P* < 0.01, ∗∗∗*P* < 0.001.

The further detailed OPLS-DA analysis visualized the optimal separation between the DKD group and CON group in the test cohort (R^2^X = 0.510, R^2^Y = 0.910, and Q^2^ = 0.851, Fig. 2A). The SUS plot and ROC analyses showed 12-HETE, ARA, 14(S)-HDHA, 13-HDHA, 5-oxoETE, 6keto-PGF1α, 13-oxoODE, 16-HDHA, 5-HETE, and 13(S)-HpODE as key oxylipins identifying the DKD group from the CON group with optimal discrimination and predictive ability (Fig. 2B, C). The OPLS-DA analysis also visualized the separation between the DKD group and T2DM group (R^2^X = 0.377, R^2^Y = 0.831, and Q^2^ = 0.729, Fig. 2D). The SUS plots and ROC analysis identifying 13-oxoODE, 8-HETE, 14(S)-HDHA, 17-HDHA, 15(S)-HETrE, 5-HETE, 14,15-EET, 8(S)-HETrE, 11-HEDE, 10-HDHA, 8-HDHA, 13(S)-HpODE, 12-HETE, 8,9-EET, 5-oxoETE, 11,12-EET, ARA, 13-HDHA, 11-HDHA, 15-HEDE, and 16-HDHA as key oxylipins discriminating the DKD group from T2DM group (Fig. 2E, F). In addition, it also indicated that γ-linoleic acid, ARA, 5-HETE, 6keto-PGF1α, 5-oxoETE, 13-oxoODE, 12-HETE, 14(S)-HDHA, and 13(S)-HpODE were the key oxylipins discriminating the DKD group from the non-DKD group (either CON or T2DM, R^2^X = 0.245, R^2^Y = 0.793, and Q^2^ = 0.736, Fig. 2G–I), whereas 5-oxoETE, 8-HDHA, 9-HETE, ARA, 14(15)-EpETE, 15-HETE, 5-HETE, 6keto-PGF1α, 13-HDHA, 16-HDHA, 7-HDHA, 8-HETE, 4-HDHA, 11-HDHA, 11(S)-HETE, and 5-HEPE were the key oxylipins identifying the patients in diabetic condition (either T2DM or DKD) from the CON group (R^2^X = 0.415, R^2^Y = 0.883, and Q^2^ = 0.795, Fig. 2J–L). Accompanied by the significant increase of ARA, 5-HETE, and 5-oxoETE in the DKD group than either the T2DM group or the CON group (Fig. 2M–O), these results indicated the possible association of the abnormal activation of ARA/5-HETE/5-oxoETE metabolism with the progression of DKD.

### The separated oxylipin profiling in the validation cohort

**Fig. 3:**
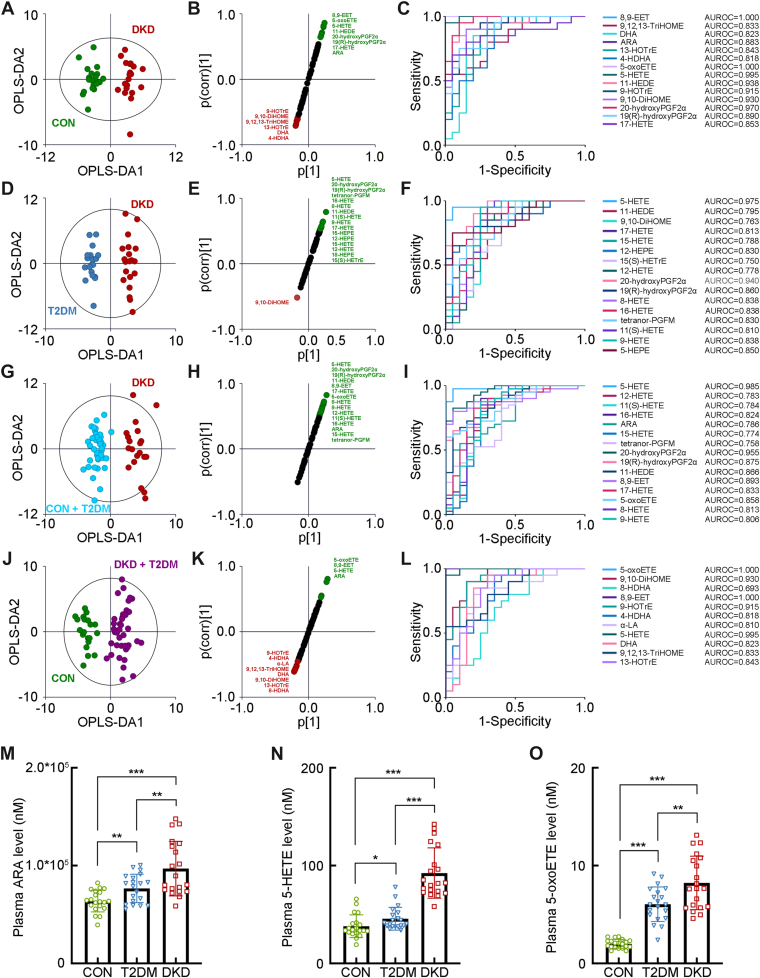
The separated metabolomic profiling in the second validation cohort. A: The OPLS-DA score plot between the DKD group and the CON group. The SUS plot (B) and ROC curve analyses (C) of the key oxylipins between the DKD group and the CON group. D: The OPLS-DA score plot between the DKD group and the T2DM group. The SUS plot (E) and ROC curve analyses (F) of the key oxylipins between the DKD group and the T2DM group. G: The OPLS-DA score plot between the DKD group and the non-DKD group (both the T2DM group and the CON group). The SUS plot (H) and ROC curve analyses (I) of the key oxylipins between the DKD group and the non-DKD group. J: The OPLS-DA score plot between the group in diabetic condition (both DKD and T2DM groups) and the CON group. The SUS plot (K) and ROC curve analyses (L) of the key oxylipins between the group in diabetic condition and the CON group. The levels of the key fecal bile acids, including (M) ARA, (N) 5-HETE, and (O) 5-oxoETE. ∗*P* < 0.05, ∗∗*P* < 0.01, and ∗∗∗*P* < 0.001.

The separated OPLS-DA analysis of the second validation cohort showed the optimal separation between the DKD group and the CON group (R^2^X = 0.295, R^2^Y = 0.914, and Q^2^ = 0.787, Fig. 3A), with 8,9-EET, 9,12,13-TriHOME, DHA, ARA, 13-HOTrE, 4-HDHA, 5-oxoETE, 5-HETE, 11-HEDE, 9-HOTrE, 9,10-DiHOME, 20-hydroxyPGF2α, 19(R)-hydroxyPGF2α, and 17-HETE as the key oxylipins to group difference (Fig. 3B, C). The OPLS-DA analysis between the DKD group and the T2DM group (R^2^X = 0.686, R^2^Y = 0.962, and Q^2^ = 0.731, Fig. 3D) also showed the ideal modeling with 5-HETE, 11-HEDE, 9,10-DiHOME, 17-HETE, 15-HETE, 12-HEPE, 15(S)-HETrE, 12-HETE, 20-hydroxyPGF2α, 19(R)-hydroxyPGF2α, 8-HETE, 16-HETE, tetranor-PGFM, 11(S)-HETE, 9-HETE, and 5-HEPE as the key oxylipins contributing to the group difference (Fig. 3E, F). In addition, it also indicated that 5-HETE, 12-HETE, 11(S)-HETE, 16-HETE, ARA, 15-HETE, tetranor-PGFM, 20-hydroxyPGF2α, 19(R)-hydroxyPGF2α, 11-HEDE, 8,9-EET, 17-HETE, 5-oxoETE, 8-HETE, and 9-HETE as the key oxylipins discriminating the DKD group from the non-DKD group (R^2^X = 0.545, R^2^Y = 0.904, and Q^2^ = 0.774, Fig. 3G–I), whereas 5-oxoETE, 9,10-DiHOME, 8-HDHA, 8,9-EET, 9-HOTrE, 4-HDHA, α-linoleic acid, 5-HETE, DHA, 9,12,13-TriHOME, and 13-HOTrE were the key oxylipins identifying the patients in diabetic condition from the CON group (R^2^X = 0.623, R^2^Y = 0.875, and Q^2^ = 0.703, Fig. 3J–L). Combing with the significant increase of ARA, 5-HETE, and 5-oxoETE in the DKD group, the results in the validation cohort, in consistent with those in the test cohort (Fig. 3M–O), indicated the strong association between the activated ARA/5-HETE/5-oxoETE metabolism and the progression of DKD.

### The correlation matrix of oxylipins

**Fig. 4:**
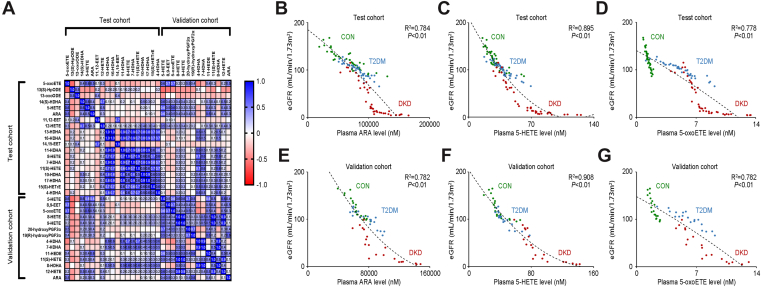
Correlation matrix for the oxylipins in the study participants. A: The correlation heat map of the key oxylipins in the study participants of the test cohort and validation cohort. The blue area shows strong positive correlation, and the red area shows strong negative correlation. The specific Pearson’s *r* value is presented in each cell. The cells in white were defined as *R* = 0. Scatter plots show the correlation of eGFR with the plasma levels of ARA (B), 5-HETE (C), and 5-oxoETE (D) in the test cohort as well as those in the validation cohort (E–G).

The correlation matrix for oxylipins in study participants was performed, showing the positive correlation of the key oxylipins between the test and validation cohorts (Fig. 4A). ARA, 5-HETE, and 5-oxoETE in the test cohort are correlated with 5-HETE and 5-oxoETE but not with ARA in the validation cohort. When analyzing their correlation with the clinical indicators reflecting DKD progression, including hemoglobin, serum albumin, serum creatinine, blood glucose, serum triglyceride, total cholesterol, low-density lipoprotein, high-density lipoprotein, parathyroid hormone, glycated hemoglobin, estimated glomerular filtration rate (eGFR) and urinary protein and urinary microalbumin in 24 h, it presented the strong correlation of plasma ARA (*R*^2^ = 0.784), 5-HETE (*R*^2^ = 0.895), and 5-oxoETE (*R*^2^ = 0.778) with eGFR in the test cohort (Fig. 4B–D), and consistently, the strongly correlation in the validation cohort (ARA, *R*^2^ = 0.782; 5-HETE, *R*^2^ = 0.908; and 5-oxoETE *R*^2^ = 0.782, Fig. 4E–G). These results indicated the optimal correlation of plasma ARA, 5-HETE, and 5-oxoETE, both in the test cohort and the validation cohort, with eGFR in patients with DKD.

### Validation and development of the diagnostic models

**Fig. 5:**
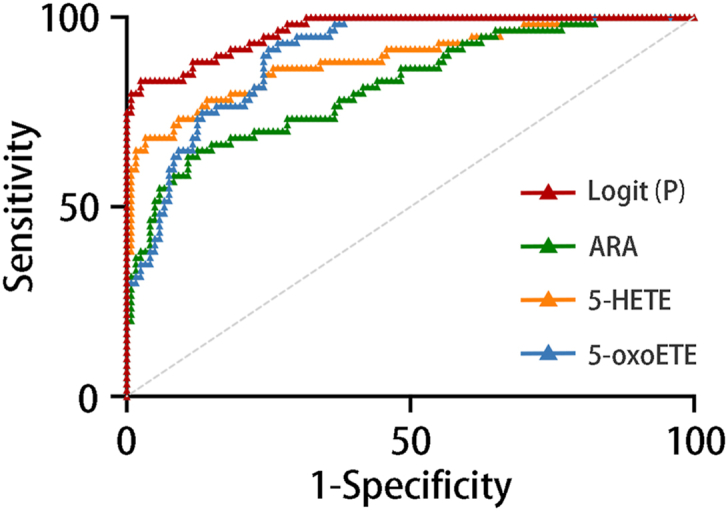
The ROC curve for the plasma ARA, 5-HETE, 5-oxoETE, and the diagnostic model of the combination of the three oxylipins. Logit (P) represents the diagnostic model of the combination of plasma ARA, 5-HETE, and 5-oxoETE. The detailed AUROC, cutoff value, sensitivity, specificity, and accuracy are presented in Table 1.

To validate the distinguishing ability of the key oxylipins, logistic regression was performed, and the diagnostic models were established on the basis of plasma levels of ARA, 5-HETE, and 5-oxoETE in the integration of both the test cohort and validation cohort (Table 1). The diagnostic regression equation was as follows: Logit (P) = 0.001 ∗ Log (ARA) + 0.076 ∗ Log (5-HETE) + 0.570 ∗ Log (5-oxoETE) - 11.895, with a cutoff value of 0.626. It showed an excellent diagnostic ability of Logit (P), with AUROC of 0.820 ± 0.034, Jordan index of 0.808, sensitivity of 97.5%, specificity of 83.3%, and accuracy of 88.0% (Fig. 5). These results indicate the optimal diagnostic performance of plasma ARA, 5-HETE, and 5-oxoETE for patients with DKD and much better performance of the combined diagnostic model.

**Table 1:** Predictive performance of the key oxylipins and the combined diagnostic model

Oxylipin	AUC	*P*^a^	Cutoff	Jordan index	Sensitivity (%)	Specificity (%)	Accuracy (%)
ARA	0.820 ± 0.034	<0.001	95,462.6 nM	0.525	87.5	65.0	72.5
5-HETE	0.890 ± 0.028	<0.001	63.688 nM	0.650	96.7	68.3	77.8
5-oxoETE	0.901 ± 0.022	<0.001	5.377 nM	0.667	73.3	93.3	86.6
Logit (P)	0.965 ± 0.012	<0.001	0.626	0.808	97.5	83.3	88.0

Accuracy = (A × sensitivity + B × specificity)/(A + B), where A is the number of participants in the corresponding disease group and B is the number of participants in the corresponding control group.

a *P* values were determined by analyses of ROC curves under a nonparametric assumption.

## Discussion

Over decades, considerable effort has been made to elucidate the molecular factors responsible for diabetes as well as its complications. Whilst severe lipid metabolism disorders, including oxylipins, are raised to accompany diabetes, it is still uncertain the exact alterations of oxylipin metabolism in T2DM, and much less concern for DKD (19). To date, conflicting results exist regarding the effects of oxylipins in diabetes. Specifically, ω-3-derived oxylipins are found to have generally protective effects, whereas oxylipins originating from ω-6 PUFAs have detrimental roles (20). The pleiotropic effects of these compounds are increasingly appreciated, with different oxylipins having dissimilar roles in physiological and pathophysiological processes. Thus, profiling of oxylipins can be an important tool in investigating their role in the pathogenesis of T2DM as well as DKD.

Our study revealed a significant activation of ARA/5-HETE metabolism in patients with DKD. 5-HETE is a proinflammatory eicosanoid oxidized from ARA by iron-containing lipoxygenase ALOX5. The activation of ARA/5-HETE metabolism depends not only on the activation of ALOX5 but also requires a stimulus of cytosolic NADP^+^ and thus is associated with both inflammation and oxidative stress (21). As ALOX5 widely exists in different organs and tissues, 5-HETE plays a significant role in normal physiological and pathophysiological conditions, notably causing beta cells and endothelial cell dysfunction, as well as regulating the inflammation in adipose tissue (22). It is still uncertain whether the alterations of ARA/5-HETE metabolism are the result or the cause of DKD. A study in a small sample size, consistent with our findings, showed a significant increase in plasma 5-HETE in patients with DKD than those in patients with T2DM alone, yet not identifying the oxylipin profiling in healthy individuals (23). Some other studies have also reported the increased activation of ALOX5 in the blood cells from dogs with acute kidney injury, chronic kidney disease, and the uremic patients undergoing maintenance hemodialysis (24, 25). This phenomenon is probably mediated by complement activation and may be an important proinflammatory event leading to capillary wall injury in the early heterologous phase. However, decreasing plasma 5-HETE by supplying n-3 PUFA seems to have no improvement on kidney function, implicating the possible far more proinflammatory role of the metabolite of 5-HETE (16). Nevertheless, a study exploring the oxylipin profiling in uremic patients presented a significant decrease in plasma 5-HETE and inactivation of ALOX5 in the whole blood cells (14). Thus, there is a lack of relevant studies focusing specifically on the causality between ALOX5-mediated ARA/5-HETE metabolism and kidney disease.

Apart from the kidney, accumulated evidence has shown the pleiotropic effects of ARA/5-HETE metabolism in inflammation, insulin resistance, and diabetes. 5-HETE is one of a series of proinflammatory lipid mediators that are catalyzed by ALOX5 in adipose tissue and are responsible for chronic low-grade inflammation and insulin resistance status (26). Pharmaceutical approaches targeting ALOX5 or 5-HETE have been shown to be anti-inflammatory via reducing adipose tissue macrophage infiltration, decreasing M1:M2 phenotype, and lowering free fatty acid release (27, 28). 5-HETE produced by adipose tissue can be further released systematically to stimulate inflammation in other tissues, including kidney (29). Research has also demonstrated that ARA/5-HETE metabolism is associated with inflammation across multiple organs, with its metabolite 5-HETE promoting chronic inflammation through the upregulation of proinflammatory cytokines, such as IL-1β and TNF-α, potentially leading to the deterioration of kidney function (30). Thus, the inflammation and insulin resistance, which is induced by the aberrant activation of ARA/5-HETE metabolism, may represent a primary mechanism underlying DKD progression.

Aside from the inflammation and insulin resistance, it may also be attributed to the ALOX5-mediated ferroptosis. Ferroptosis, different from apoptosis, necrosis, and autophagy, is a type of programmed cell death driven by iron-dependent lipid peroxidation. Lipid peroxidation is central to the mechanism of ferroptosis, with ARA and its metabolites playing a key role in promoting this process. The increased PUFA metabolism results in the accumulation of ARA on the cell membrane, leading to the activation of ARA/5-HETE metabolism catalyzed by ALOX5, and thereafter the destruction of the lipid bilayer of the cell membrane as well as initiation of cell ferroptosis (31). A previous study has implicated that the aberrant activation of ARA/5-HETE metabolism could accelerate DKD progression by inducing oxidative stress. The accumulation of 5-HETE and related metabolites correlates with increased reactive oxygen species production, with oxidative stress being a critical contributor to renal fibrosis and functional decline (32). The oxidative damage may thus exacerbate tubulointerstitial fibrosis, and thereafter, the renal function deterioration in patients with DKD. In addition, the disruption of ARA/5-HETE metabolism could compromise lipid metabolism, damaging the integrity of tubular epithelial cell membranes, promoting lipid peroxidation, and eventually leading to cell death (33). Thus, although there is no direct evidence indicating the activation of ALOX5-mediated ARA/5-HETE metabolism in the renal tissue of DKD patients, the aforementioned studies implied the strong association of ALOX5-ARA/5-HETE-mediated ferroptosis in the renal tubular injury in patients with DKD.

In clinical practice, patients with DKD usually exhibit levels of blood glucose that are similar to those patients with T2DM alone. At present, the gold standard in diagnosing DKD is the histological feature based on invasive renal biopsy. The severity of proteinuria and eGFR accompanied by diabetes-related comorbidities is also the recommended clinical diagnosis standard (34). However, as the renal biopsy is an invasive procedure that can be complicated by hematuria or perirenal hematoma, whilst the accuracy of proteinuria and eGFR can be interfered with by various factors. Moreover, substantial kidney damage may occur before the abnormality of eGFR or proteinuria. Notably, our metabolomic study presented excellent performance of the key differential oxylipins, more specifically of plasma ARA, 5-HETE, and 5-oxoETE, distinguishing DKD from T2DM and healthy individuals without consideration of the value changes in the proteinuria and eGFR range, through less invasive clinical practice. In addition, the positive correlation of plasma ARA, 5-HETE, and 5-oxoETE both in the test cohort and the validation cohort, as well as the strong correlation of plasma ARA, 5-HETE, and 5-oxoETE with eGFR, which commonly reflects renal function, highlights the association between the enhanced activation of ARA/5-HETE metabolism and the progression of DKD. To further explore their clinical potential, the diagnostic models were established on the basis of plasma levels of ARA, 5-HETE, and 5-oxoETE in the integration of both the test cohort and validation cohort. The AUROC of our diagnostic model was 0.965 in the corresponding ROC curve, with high specificity, sensitivity, and accuracy, indicating excellent diagnostic performance in identifying DKD.

Some limitations exist regarding our study. First, as a cross-sectional study, there is insufficient evidence explicating the causality between oxylipin profiles and DKD progression. Second, the sample size of our study was relatively small; the oxylipin profiles at different stages of DKD were not sufficiently determined, particularly the nonalbuminuric phenotype. Third, since our targeted oxylipin metabolomic approach focused on specific oxylipins, a combination of untargeted and targeted metabolomic research could furnish a more comprehensive metabolic profile and reveal potentially unrecognized metabolic changes along with the progression of DKD. Therefore, further investigations, including the prospective longitudinal, *in vivo*, or *in vitro* intervention studies, are needed to explore the association between ARA/5-HETE metabolism and the progression of DKD.

Our study preliminarily presented the markedly step-wise alterations of plasma oxylipins in patients with DKD, highlighting the aberrant activation of the ARA/5-HETE metabolism and its association with the decline of renal function. In addition, our findings provided a theoretical basis regarding the combination of plasma levels of ARA, 5-HETE, and 5-oxoETE as a specific, real-time, and simple biomarker for the diagnosis of DKD. Although further mechanistic studies are required, our findings may provide the basis not only for the early identification but also for the therapeutic strategies for DKD.

## Data availability

The raw data generated or analyzed during this study are available from the corresponding author on reasonable request.

## Supplemental data

This article contains supplemental data.

## Conflict of interest

The authors declare that they have no conflicts of interest with the contents of this article.
