# Supplementary material for: Plasma oxylipin profiling reveals the step-wise activation of ARA/5-HETE metabolism in diabetic kidney disease
Source: J Lipid Res. 2025 Oct 1;67(3):100918. doi: 10.1016/j.jlr.2025.100918 (PMC12936733; doi:10.1016/j.jlr.2025.100918)
Supplement: Supplementary Tables and Figure [file mmc1.docx]

**Supplementary materials for**

**Figure and Table of Contents**

**Supplemental Fig. S1.** The validation of the OPLS-DA models.

**Supplemental Table S1.** Gradient program of the liquid chromatography mobile phase.

**Supplemental Table S2.** The optimized mass spectrometry conditions for the detection of oxylipins.

**Supplemental Table S3.** The identification and mass spectrometry parameters of oxylipins in our project.

**Supplemental Table S4.** The mass spectrometry parameters of the isotoped-labelled oxylipins.

**Supplemental Table S5.** Demographic characteristics of the study participants.

**Supplemental Table S6.** Plasma levels of the detected oxylipins in the patients of test cohort.

**Supplemental Table S7.** Plasma levels of the detected oxylipins in the patients of validation cohort.

**
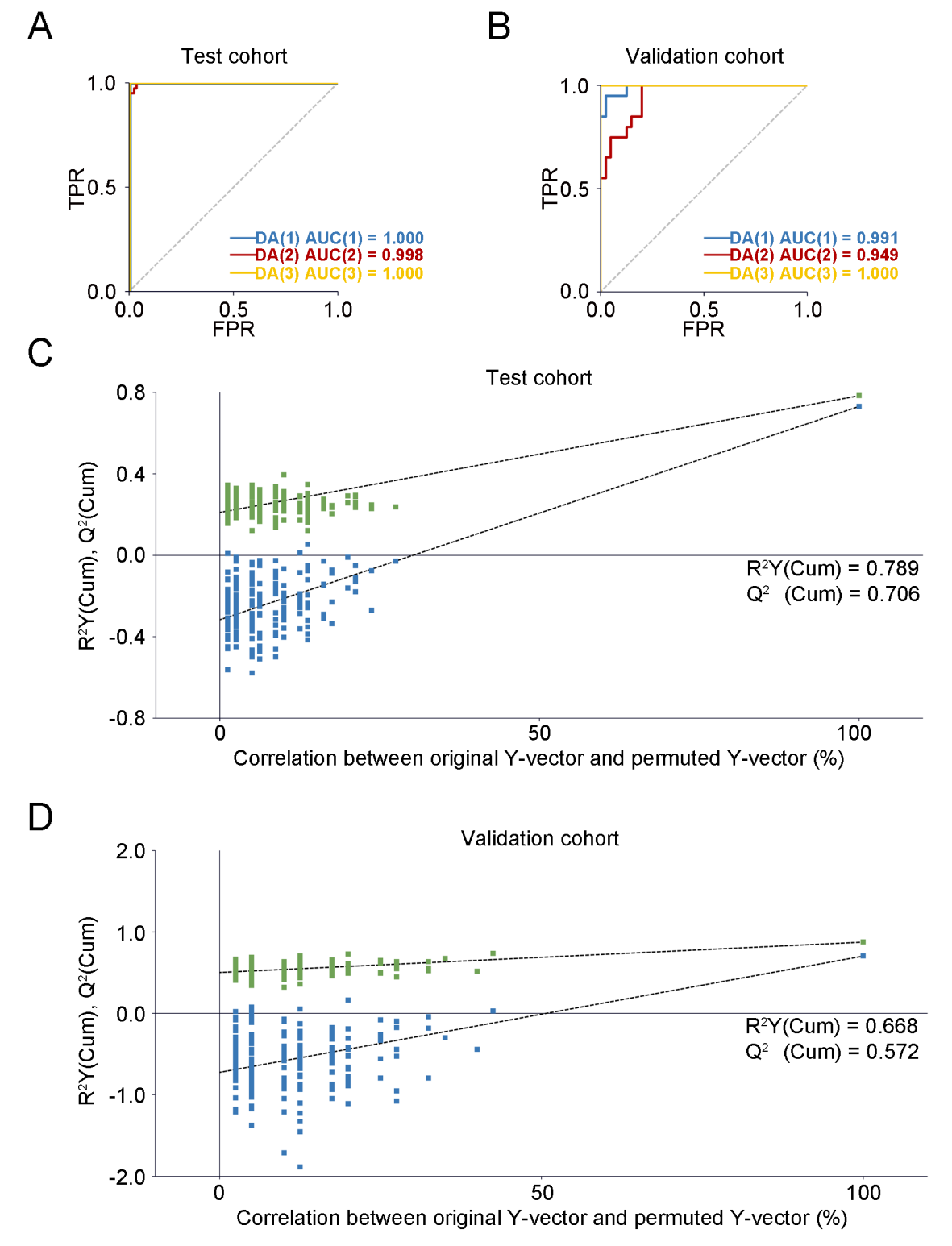
**

**Supplemental Fig. S1. The validation of the OPLS-DA models.** The ROC analyses of the binary classifier of the OPLS-DA models in the **(A)** test cohort and **(B)** validation cohort. The permutation plots of the OPLS-DA models in the **(D)** test cohort and **(E)** validation cohort. The permutation indicates the correlation coefficient between the original R^2^(green dots), Q^2^ (blue dots), and cumulative R^2^, Y^2^; the dashed lines represent the corresponding regression lines. The number of random permutation tests for each plot was 200. OPLS-DA: Orthogonal partial-least-squares discriminant analysis; ROC: receiver operator characteristic; AUC: area under the ROC curve; TPR: ture positive rate; FPR: false positive rate.

**Supplemental Table S1.** Gradient program of the liquid chromatography mobile phase.

| **Total time (min)** | **Flow rate (μL/min)** | **A^a^ (v/v, %)** | **B^b^ (v/v, %)** |
| --- | --- | --- | --- |
| 0.0 | 400 | 99.9 | 0.1 |
| 2.0 | 400 | 70 | 30 |
| 4.0 | 400 | 50 | 50 |
| 5.5 | 400 | 1 | 99 |
| 7.0 | 400 | 1 | 99 |
| 7.1 | 400 | 99.9 | 0.1 |

^a^Solvent A, acetonitrile/water (60/40, v/v) containing 0.04% acetic acid (v/v); ^b^Solvent B, acetonitrile/isopropanol (50/50, v/v). Temperature, 40℃; injection volume, 10 μL.

**Supplemental Table S2.** The optimized mass spectrometry conditions for the detection of oxylipins.

| Item | Parameter |
| --- | --- |
| Capillary voltage | -4500V |
| Source temperature | 550 °C |
| Curtain gas | 35 psi |
| Collision gas | 10 psi |
| Pressure for nebulization gas | 40 psi |
| Evaporization gas | 40 psi |
| Entrance potential | 50 V |
| Collision cell exit | 10 V |

**Supplemental Table S3.** The identification and mass spectrometry parameters of oxylipins in our project.

| No. | Compounds | CAS | MRM  transition | Linearity  (R2) | RT  (min) | Range  (nM) | Intra-day precision (%) | Inter-day precision (%) |
| --- | --- | --- | --- | --- | --- | --- | --- | --- |
| 1 | 10-HDHA | 90780-50-0 | 343.2→153 | 0.9987 | 3.39 | 1→4000 | 3% | 11% |
| 2 | 11(12)-DiHET | 192461-95-3 | 337→167.1 | 0.9994 | 2.35 | 0.2→400 | 4% | 4% |
| 3 | 11(12)-DiHETE | 867350-92-3 | 335.1→167.1 | 0.9998 | 1.85 | 0.2→400 | 4% | 4% |
| 4 | 11(S)-HETE | 54886-50-9 | 319.1→167.3 | 0.9980 | 3.40 | 2→4000 | 10% | 8% |
| 5 | 11,12-EET | 81276-02-0 | 319.3→179 | 0.9991 | 4.19 | 0.2→400 | 2% | 27% |
| 6 | 11-deoxyPGE1 | 37786-00-8 | 337.2→319.3 | 0.9964 | 1.67 | 0.2→400 | 8% | 10% |
| 7 | 11-HDHA | 87018-59-5 | 343.2→149.2 | 0.9936 | 3.48 | 1→4000 | 3% | 9% |
| 8 | 11-HEDE | 5598-37-8 | 323.5→199.1 | 0.9968 | 4.16 | 0.2→400 | 4% | 6% |
| 9 | 11-HEPE | 99217-78-4 | 317→255 | 0.9956 | 2.76 | 1→400 | 9% | 9% |
| 10 | 11-keto-TXB2 | 67910-12-7 | 367.3→161.1 | 0.9901 | 1.00 | 0.2→400 | 22% | 18% |
| 11 | 11-transLTE4 | 75715-88-7 | 438.3→235.2 | 0.9914 | 1.19 | 0.2→400 | 19% | 15% |
| 12 | 11β-PGE2 | 38310-90-6 | 351.3→271.3 | 0.9956 | 1.00 | 0.4→400 | 4% | 10% |
| 13 | 11β-PGF2α | 38432-87-0 | 353.2→309.4 | 0.9969 | 0.84 | 1→400 | 2% | 4% |
| 14 | 12(13)-DiHOME | 263399-35-5 | 313→183 | 0.9993 | 1.90 | 0.2→400 | 5% | 4% |
| 15 | 12,13-EpOME | - | 295.2→195.1 | 0.9992 | 3.97 | 0.2→400 | 9% | 7% |
| 16 | 12-epiLTB4 | 83709-73-3 | 335→195 | 0.9991 | 1.72 | 1→400 | 8% | 12% |
| 17 | 12-HEPE | 81187-21-5 | 317.2→179.2 | 0.9991 | 2.89 | 0.2→400 | 6% | 7% |
| 18 | 12-HETE | 71030-37-0 | 319.3→179 | 0.9975 | 3.53 | 2→4000 | 13% | 11% |
| 19 | 12-HHT | 54397-84-1 | 279.2→217.2 | 0.9978 | 2.18 | 0.4→400 | 9% | 8% |
| 20 | 12-oxo-ETE | 108437-64-5 | 317→153 | 0.9949 | 3.54 | 4→400 | 5% | 7% |
| 21 | 13(14)-DiHDPE(A) | 1345275-24-2 | 361.5→193.1 | 0.9995 | 2.34 | 0.2→400 | 1% | 3% |
| 22 | 13(S)-HODE | 29623-28-7 | 295.1→195.1 | 0.9995 | 3.12 | 0.2→400 | 4% | 3% |
| 23 | 13(S)-HOTrE(γ) | 74784-20-6 | 293→193 | 0.9974 | 2.67 | 0.2→400 | 4% | 4% |
| 24 | 13(S)-HpODE | 33964-75-9 | 311.1→293.2 | 0.9947 | 3.25 | 4→400 | 17% | 18% |
| 25 | 13,14-dihydro-15-ketoPGD2 | 59894-07-4 | 351.2→175.2 | 0.9901 | 1.34 | 0.4→400 | 36% | 25% |
| 26 | 13,14-dihydro-15-ketoPGE2 | 363-23-5 | 351.1→175.1 | 0.9941 | 1.35 | 4→400 | 32% | 25% |
| 27 | 13,14-dihydro-15-ketoPGF2α | 27376-76-7 | 353.2→183.2 | 0.9968 | 1.17 | 4→400 | 4% | 6% |
| 28 | 13,14-dihydroPGF2α | 27376-74-5 | 355.1→311.4 | 0.9991 | 1.04 | 4→400 | 6% | 5% |
| 29 | 13-HDHA | 90780-53-3 | 343.2→221.2 | 0.9950 | 3.32 | 1→4000 | 3% | 9% |
| 30 | 13-HOTrE | 87984-82-5 | 293.2→195.1 | 0.9946 | 2.58 | 0.2→400 | 1% | 7% |
| 31 | 13-oxoODE | 54739-30-9 | 293→112.8 | 0.9955 | 3.24 | 0.2→400 | 7% | 11% |
| 32 | 14(15)-DiHET | 77667-09-5 | 337→207.2 | 0.9989 | 2.16 | 0.2→400 | 3% | 2% |
| 33 | 14(15)-DiHETE | - | 335.1→207 | 0.9983 | 1.82 | 0.2→400 | 3% | 4% |
| 34 | 14(15)-EpETE | 131339-24-7 | 317→207 | 0.9965 | 3.49 | 0.2→400 | 5% | 6% |
| 35 | 14(S)-HDHA | 87042-40-8 | 343.1→205 | 0.9968 | 3.39 | 4→4000 | 2% | 12% |
| 36 | 14,15-EET | 81276-03-1 | 319.3→219.3 | 0.9975 | 4.00 | 0.2→400 | 1% | 3% |
| 37 | 14,15-LTE4 | 1000852-57-2 | 438.1→143.9 | 0.9979 | 0.97 | 4→400 | 7% | 5% |
| 38 | 15(S)-HETrE | 13-16-1 | 321.2→221.1 | 0.9955 | 3.64 | 0.2→400 | 6% | 6% |
| 39 | 15-deoxy-12,14-PGA2 | 112839-31-3 | 315.2→271.1 | 0.9922 | 2.72 | 0.2→400 | 13% | 16% |
| 40 | 15-HEDE | 77159-57-0 | 323.5→223.1 | 0.9936 | 4.18 | 0.2→400 | 4% | 6% |
| 41 | 15-HEPE | 88852-33-9 | 317.2→219.2 | 0.9932 | 2.76 | 0.2→400 | 9% | 9% |
| 42 | 15-HETE | 71030-36-9 | 319.2→219.1 | 0.9980 | 3.26 | 2→4000 | 10% | 8% |
| 43 | 15-keto-PGE2 | 26441-05-4 | 349.4→235 | 0.9926 | 1.09 | 0.4→400 | 14% | 14% |
| 44 | 15-keto-PGF1α | 21562-58-3 | 353.3→221.2 | 0.9907 | 1.04 | 4→400 | 9% | 5% |
| 45 | 15-keto-PGF2α | 35850-13-6 | 351.4→315.1 | 0.9949 | 1.02 | 4→400 | 14% | 10% |
| 46 | 15-oxo-ETE | 81416-72-0 | 317.2→113.2 | 0.9986 | 3.45 | 0.2→400 | 6% | 8% |
| 47 | 16(17)-EpDPE | 155073-46-4 | 343.4→232.9 | 0.9986 | 4.02 | 0.2→400 | 2% | 4% |
| 48 | 16-HDHA | 90780-51-1 | 343.2→233 | 0.9982 | 3.25 | 1→4000 | 4% | 11% |
| 49 | 16-HETE | 128914-46-5 | 319→189.2 | 0.9982 | 2.99 | 0.2→400 | 11% | 12% |
| 50 | 17(18)-DiHETE | - | 335.2→247.1 | 0.9996 | 1.69 | 0.2→400 | 4% | 4% |
| 51 | 17(18)-EpETE | 131339-23-6 | 317.2→215.1 | 0.9947 | 3.32 | 0.2→400 | 8% | 6% |
| 52 | 17-HDHA | 90780-52-2 | 343→245.4 | 0.9961 | 3.29 | 4→4000 | 4% | 10% |
| 53 | 17-HETE | 128914-47-6 | 319→247 | 0.9970 | 2.96 | 0.2→400 | 12% | 11% |
| 54 | 18-HEPE | 141110-17-0 | 317.2→255.4 | 0.9980 | 2.58 | 0.4→400 | 10% | 11% |
| 55 | 18-HETE | 133268-58-3 | 318.9→261.2 | 0.9977 | 2.91 | 0.2→400 | 8% | 8% |
| 56 | 19(20)-DiHDPE(A) | - | 361→229 | 0.9987 | 2.12 | 4→400 | 3% | 3% |
| 57 | 19(20)-EpDPE(A) | - | 343→241 | 0.9962 | 3.84 | 0.2→400 | 10% | 11% |
| 58 | 19(R)-hydroxyPGF2α | 64625-53-2 | 369.1→325.3 | 0.9988 | 0.78 | 1→400 | 14% | 14% |
| 59 | 19(S)-HETE | 79551-85-2 | 319→231.2 | 0.9965 | 2.70 | 0.4→400 | 12% | 10% |
| 60 | 1-Mar | 1268720-28-0 | 359.1→250.2 | 0.9995 | 1.65 | 0.2→400 | 3% | 5% |
| 61 | 2,3-dinor-8-iso-PGF2α | 221664-05-7 | 325→237.3 | 0.9962 | 0.78 | 2→400 | 0% | 3% |
| 62 | 20-COOH-ARA | 79551-84-1 | 333.2→271.2 | 0.9949 | 2.38 | 0.4→400 |  |  |
| 63 | 20-COOH-LTB4 | 80434-82-8 | 365.1→169.2 | 0.9987 | 0.81 | 10→400 | 6% | 6% |
| 64 | 20-HDHA | 90906-41-5 | 343.2→241.2 | 0.9963 | 3.13 | 2→4000 | 5% | 12% |
| 65 | 20-HETE | 79551-86-3 | 318.8→289.2 | 0.9941 | 2.74 | 0.2→400 | 10% | 11% |
| 66 | 20-hydroxyPGF2α | 57930-92-4 | 369.3→193.1 | 0.9971 | 0.78 | 1→400 | 4% | 4% |
| 67 | 20-OH-LTB4 | 79516-82-8 | 351.1→195.1 | 0.9980 | 0.80 | 0.2→400 | 10% | 8% |
| 68 | 4-HDHA | 90906-40-4 | 343.2→100.7 | 0.9986 | 3.84 | 4→4000 | 9% | 11% |
| 69 | 5(S),12(S)-DiHETE | 79056-01-2 | 335.2→335.2 | 0.9978 | 1.92 | 1→400 | 2% | 3% |
| 70 | 5(S),15(S)-DiHETE | 82200-87-1 | 335.1→114.9 | 0.9968 | 1.64 | 0.2→400 | 6% | 6% |
| 71 | 5,6-DIHETE | 845673-97-4 | 335.4→145.2 | 0.9994 | 2.09 | 1→400 | 4% | 3% |
| 72 | 5,6-DiHETrE | 213382-49-1 | 337.2→145.1 | 0.9988 | 2.73 | 0.2→400 | 2% | 2% |
| 73 | 5,6-EET | 81246-84-6 | 319.2→191.2 | 0.9995 | 4.27 | 1→400 | 5% | 6% |
| 74 | 5-HEPE | 83952-40-3 | 317.1→114.7 | 0.9943 | 3.01 | 0.2→400 | 11% | 8% |
| 75 | 5-HETE | 70608-72-9 | 319.2→115 | 0.9987 | 3.71 | 4→4000 | 2% | 2% |
| 76 | 5-HETrE | 195061-94-0 | 321.1→114.9 | 0.9993 | 4.49 | 0.2→400 | 2% | 4% |
| 77 | 5-isoPGF2VI | - | 353.2→309.3 | 0.9994 | 0.84 | 0.2→400 | 2% | 4% |
| 78 | 5-oxo-ETE | 106154-18-1 | 317.2→203.2 | 0.9993 | 4.09 | 0.2→400 | 5% | 8% |
| 79 | 6keto-PGF1α | 58962-34-8 | 369.3→163.1 | 0.9988 | 0.82 | 0.2→400 | 4% | 5% |
| 80 | 6-trans-12-epiLTB4 | 71548-19-1 | 335.1→195.1 | 0.9985 | 1.66 | 1→400 | 8% | 12% |
| 81 | 6-transLTB4 | 71652-82-9 | 335.1→195 | 0.9979 | 1.66 | 0.1→400 | 0% | 4% |
| 82 | 7(8)-DiHDPE(A) | - | 361.5→113 | 0.9991 | 2.64 | 0.2→400 | 4% | 4% |
| 83 | 7,8-EpDPE | 895127-66-9 | 343.2→343.2 | 0.9972 | 4.17 | 0.2→400 | 7% | 9% |
| 84 | 7-HDHA | 90780-55-5 | 343.1→140.9 | 0.9961 | 3.53 | 4→4000 | 3% | 8% |
| 85 | 8(9)-DiHET | 192461-96-4 | 337→127 | 0.9996 | 2.51 | 0.2→400 | 1% | 1% |
| 86 | 8(9)-DiHETE | 867350-87-6 | 335.1→127.1 | 0.9978 | 1.93 | 0.2→400 | 4% | 4% |
| 87 | 8(S),15(S)-DiHETE | 80234-65-7 | 335→155 | 0.9988 | 1.57 | 0.2→400 | 6% | 4% |
| 88 | 8(S)-HETrE | 889573-69-7 | 321.2→157.2 | 0.9967 | 3.75 | 0.2→400 | 5% | 5% |
| 89 | 8,9-EET | 81246-85-7 | 319.3→155 | 0.9993 | 4.26 | 0.2→400 | 1% | 3% |
| 90 | 8-HDHA | 90780-54-4 | 343.2→109.2 | 0.9914 | 3.59 | 1→4000 | 3% | 7% |
| 91 | 8-HEPE | 99217-77-3 | 317→161 | 0.9982 | 2.83 | 0.2→400 | 8% | 6% |
| 92 | 8-HETE | 98462-03-4 | 319→155 | 0.9991 | 3.51 | 2→4000 | 11% | 9% |
| 93 | 8-iso-PGF2α | 27415-26-5 | 353.3→309.2 | 0.9972 | 0.85 | 10→400 | 5% | 4% |
| 94 | 9(S),10(S),13(S)-TriHOME | 29907-57-1 | 329→229 | 0.9965 | 0.92 | 0.2→400 | 10% | 9% |
| 95 | 9(S),12(S),13(S)-TriHOME | 97134-11-7 | 329→171 | 0.9922 | 0.93 | 0.4→400 | 15% | 16% |
| 96 | 9(S)-HpOTrE | 111004-08-1 | 309.2→291.2 | 0.9962 | 2.58 | 0.2→400 | 14% | 14% |
| 97 | 9,10-DiHOME | 263399-34-4 | 313.2→201 | 0.9996 | 2.00 | 0.2→400 | 3% | 3% |
| 98 | 9,10-EpOME | 16833-56-0 | 295.2→171.2 | 0.9994 | 4.03 | 0.2→400 | 3% | 4% |
| 99 | 9-HEPE | 286390-03-2 | 317→149 | 0.9982 | 2.92 | 0.2→400 | 5% | 8% |
| 100 | 9-HETE | 79495-85-5 | 319.3→150.9 | 0.9947 | 3.62 | 2→4000 | 6% | 16% |
| 101 | 9-HODE | 98524-19-7 | 295.1→277.3 | 0.9984 | 3.13 | 0.2→400 | 5% | 8% |
| 102 | 9-HOTrE | 89886-42-0 | 293→171 | 0.9965 | 2.47 | 0.2→400 | 5% | 5% |
| 103 | 9-oxo-ODE | 54232-59-6 | 293→185.1 | 0.9954 | 3.26 | 0.2→400 | 6% | 6% |
| 104 | 9-oxo-OTrE | 125559-74-2 | 291.2→185.1 | 0.9952 | 2.87 | 0.2→400 | 12% | 12% |
| 105 | ARA | 506-32-1 | 303.1→259.3 | 0.9994 | 5.46 | 40→40000 | 4% | 9% |
| 106 | DHA | 6217-54-5 | 327.2→283.3 | 0.9958 | 5.33 | 40→40000 | 16% | 13% |
| 107 | D-γ-LA | 1783-84-2 | 305.2→287.2 | 0.9978 | 5.67 | 40→40000 | 10% | 12% |
| 108 | EPA | 10417-94-4 | 300.9→257.4 | 0.9985 | 4.97 | 40→40000 | 7% | 8% |
| 109 | LA | 60-33-3 | 279.3→261.3 | 0.9986 | 5.53 | 40→40000 | 9% | 7% |
| 110 | LTB4 | 71160-24-2 | 335.1→195 | 0.9983 | 1.72 | 0.2→400 | 2% | 4% |
| 111 | LTD4 | 73836-78-9 | 495.3→142.9 | 0.9993 | 1.05 | 0.2→400 | 3% | 4% |
| 112 | LTE4 | 75715-89-8 | 438→235.2 | 0.9965 | 1.19 | 1→400 | 9% | 10% |
| 113 | LXA4 | 89663-86-5 | 351→114.9 | 0.9984 | 1.09 | 0.2→400 | 6% | 6% |
| 114 | LXA5 | 110657-98-2 | 349.2→233.1 | 0.9955 | 0.96 | 0.2→400 | 6% | 5% |
| 115 | LXB4 | 98049-69-5 | 351.2→128.9 | 0.9970 | 0.97 | 0.4→400 | 4% | 4% |
| 116 | PDX | 871826-47-0 | 359→153.3 | 0.9992 | 1.65 | 0.2→400 | 1% | 2% |
| 117 | PGA2 | 13345-50-1 | 333.1→235.2 | 0.9934 | 1.49 | 0.4→400 | 6% | 12% |
| 118 | PGB2 | 13367-85-6 | 333→271 | 0.9907 | 1.41 | 0.4→400 | 7% | 12% |
| 119 | PGD1 | 17968-82-0 | 353.1→235.2 | 0.9947 | 0.99 | 0.2→400 | 8% | 16% |
| 120 | PGD2 | 41598-07-6 | 351→233.1 | 0.9906 | 1.01 | 0.2→400 | 6% | 9% |
| 121 | PGD3 | 71902-47-1 | 349.1→189.1 | 0.9913 | 0.95 | 0.4→400 | 9% | 8% |
| 122 | PGE1 | 745-65-3 | 353.2→317 | 0.9967 | 0.99 | 0.2→400 | 6% | 10% |
| 123 | PGE2 | 363-24-6 | 351.4→271.2 | 0.9945 | 1.01 | 0.2→400 | 4% | 10% |
| 124 | PGF1α | 745-62-0 | 355.2→211 | 0.9995 | 0.91 | 0.2→400 | 13% | 9% |
| 125 | PGF2α | 551-11-1 | 353.3→192.9 | 0.9937 | 1.03 | 0.2→400 | 5% | 7% |
| 126 | PGF3α | 745-64-2 | 351.1→307.3 | 0.9926 | 0.84 | 10→400 | 7% | 4% |
| 127 | PGJ2 | 60203-57-8 | 333.2→189.1 | 0.9937 | 1.34 | 0.4→400 | 15% | 14% |
| 128 | PGK1 | 69413-73-6 | 351→251 | 0.9977 | 0.93 | 0.2→400 | 1% | 4% |
| 129 | RvD1 | 872993-05-0 | 375.3→141 | 0.9989 | 1.11 | 0.2→400 | 4% | 5% |
| 130 | RvD2 | 810668-37-2 | 375.2→215.3 | 0.9987 | 1.01 | 0.2→400 | 8% | 6% |
| 131 | RvD3 | 916888-47-6 | 375.1→147.2 | 0.9980 | 0.98 | 0.2→400 | 5% | 5% |
| 132 | RvD5 | 578008-43-2 | 359.2→199.3 | 0.9944 | 1.68 | 0.2→400 | 11% | 10% |
| 133 | RvE1 | 552830-51-0 | 349.1→161.1 | 0.9984 | 0.80 | 0.2→400 | 14% | 11% |
| 134 | tetranor-12(S)-HETE | 121842-79-3 | 265→109 | 0.9979 | 2.13 | 0.2→400 | 13% | 12% |
| 135 | tetranor-PGFM | 23109-94-6 | 329.1→293.3 | 0.9996 | 0.94 | 0.4→400 | 9% | 8% |
| 136 | trans-EKODE-(E)-Ib | 478931-82-7 | 309.2→291.2 | 0.9964 | 2.59 | 0.2→400 | 15% | 13% |
| 137 | TXB1 | 64626-32-0 | 371.1→171.1 | 0.9955 | 0.80 | 0.2→400 | 22% | 19% |
| 138 | TXB2 | 54397-85-2 | 369.1→168.8 | 0.9917 | 0.85 | 1→400 | 13% | 47% |
| 139 | TxB3 | 71953-80-5 | 367.2→169 | 0.9943 | 0.91 | 1→400 | 7% | 13% |
| 140 | α-LA | 463-40-1 | 277.2→233.1 | 0.9959 | 5.08 | 40→40000 | 7% | 15% |
| 141 | γ-LA | 506-26-3 | 277.2→233 | 0.9945 | 5.16 | 40→40000 | 6% | 13% |

**Supplemental Table S4.** The mass spectrometry parameters of the isotoped-labelled oxylipins.

| Compound | Exact mass (Da) | Q1 Mass (Da) | Q3 Mass (Da) |
| --- | --- | --- | --- |
| d4-9s-HODE | 300.5 | 299.3 | 172.1 |
| d4-LTB4 | 340.5 | 339.3 | 197.1 |
| d8-5S-HETE | 328.5 | 327.4 | 115.9 |
| d11-11,12-EET | 331.5 | 330.2 | 167 |
| (d4) PGE1 | 358.5 | 357.1 | 321.2 |
| (d7) 5-oxo-ETE | 325.5 | 324.3 | 210 |
| RvD1-d5 | 381.5 | 380.4 | 141 |
| d11-11,12-DiHETrE | 349.6 | 348.3 | 167.1 |
| 8-epi PGF2α-d4 | 358.5 | 357.3 | 251.3 |
| PGE2-d4 | 356.5 | 355.3 | 275.2 |
| PGF1α-d9 | 365.6 | 364.2 | 320.3 |
| PGF2α-d9 | 363.5 | 362.4 | 193 |
| TXB2-d4 | 374.5 | 373.3 | 173 |
| (±)14(15)-EET-d11 | 331.5 | 330.1 | 219.1 |
| LA-d11 | 291.5 | 290.2 | 272.2 |
| 9-oxo-ODE-d3 | 297.5 | 296.2 | 186.1 |
| 13-oxo-ODE-d3 | 297.5 | 296.4 | 113.9 |
| RvD3-d5 | 381.5 | 380.3 | 152.2 |
| Mar 1-d5 | 365.5 | 364.2 | 177 |
| LTD4-d5 | 501.7 | 500.1 | 177 |
| LTE4-d5 | 444.6 | 443.1 | 338.1 |
| (±)12(13)-EpOME-d4 | 300.5 | 299 | 198 |
| (±)9(10)-DiHOME-d4 | 318.5 | 317.3 | 202.9 |
| EPA-d5 | 307.5 | 306.3 | 262.1 |
| DHA-d5 | 333.5 | 332.3 | 288.2 |

**Supplemental Table S5.** Demographic characteristics of the study participants.

|  | Test cohort | | | |  | Validation cohort | | | |
| --- | --- | --- | --- | --- | --- | --- | --- | --- | --- |
|  | CON  (n=40) | T2DM  (n=40) | DKD  (n=40) | *P* |  | CON  (n=20) | T2DM  (n=20) | DKD  (n=20) | *P* |
| Age, years | 53.88±6.60 | 57.30±10.30 | 56.90±12.00 | 0.243 |  | 53.20±6.47 | 56.35±9.70 | 55.05±11.79 | 0.582 |
| Male/Female, n | 23/17 | 24/16 | 20/20 | 0.645 |  | 8/12 | 11/9 | 11/9 | 0.549 |
| Hb, g/L | 146.6±11.48 | 131.9±14.92 | 97.88±23.71 | <0.001 |  | 145.3±8.45 | 134.2±15.39 | 98.38±22.66 | <0.001 |
| Glu, mM | 4.67±0.37 | 8.98±3.18 | 6.56±3.00 | <0.001 |  | 4.53±0.41 | 8.81±2.94 | 6.76±2.73 | <0.001 |
| Alb, g/L | 49.76±3.14 | 41.07±4.20 | 33.57±7.56 | <0.001 |  | 45.80±2.95 | 41.81±3.14 | 35.89±7.30 | <0.001 |
| Scr, μM | 64.93±9.01 | 62.58±15.77 | 407.1±297.1 | <0.001 |  | 64.40±9.61 | 62.53±16.22 | 348.8±315.5 | <0.001 |
| TC, mM | 4.36±0.56 | 4.10±1.00 | 5.10±1.46 | <0.001 |  | 4.55±0.42 | 4.08±1.48 | 5.25±1.77 | 0.029 |
| TG, mM | 1.13±0.48 | 1.78±1.09 | 1.66±0.82 | 0.022 |  | 1.07±0.29 | 1.55±1.01 | 1.71±0.85 | 0.031 |
| HDL-C, mM | 1.33±0.38 | 1.05±0.30 | 1.15±0.38 | 0.003 |  | 1.34±0.29 | 1.03±0.34 | 1.00±0.17 | <0.001 |
| LDL-C, mM | 2.24±0.73 | 2.47±0.86 | 3.20±1.44 | <0.001 |  | 1.89±0.83 | 2.36±0.75 | 2.72±1.34 | 0.039 |
| eGFR, mL/min/1.73m^2^ | 121.3±20.02 | 100.2±14.44 | 38.38±36.28 | <0.001 |  | 123.3±22.77 | 100.2±17.22 | 40.31±32.00 | <0.001 |
| PTH, pg/mL | / | 30.20±13.82 | 111.5±83.83 | <0.001 |  | / | 33.80±11.30 | 131.1±126.1 | 0.001 |
| HbAlc, % | / | 6.85±1.05 | 9.06±2.02 | <0.001 |  | / | 7.37±1.20 | 9.74±2.44 | <0.001 |
| Urinary protein, g/24h | / | 0.11±0.03 | 5.44±3.39 | <0.001 |  | / | 0.11±0.03 | 4.46±3.07 | <0.001 |
| Urinary albumin, mg/24h | / | 16.15±12.45 | 2215.0±1659.0 | <0.001 |  | / | 17.37±11.59 | 1075±722.4 | <0.001 |
| Comorbidity |  |  |  |  |  |  |  |  |  |
| CAD, n | 0 | 8 | 19 | 0.009 |  | 0 | 3 | 10 | 0.018 |
| HTN, n | 0 | 17 | 35 | <0.001 |  | 0 | 8 | 14 | 0.057 |
| DR, n | 0 | 14 | 21 | 0.11 |  | 0 | 5 | 11 | 0.053 |
| DPN, n | 0 | 9 | 19 | 0.019 |  | 0 | 6 | 13 | 0.027 |

**Supplemental Table S6.** Plasma levels of the detected oxylipins in the patients of test cohort.

| Oxylipins (nM) | CON  (n=40) | T2DM  (n=40) | DKD  (n=40) | F | *P* | VIP  value |
| --- | --- | --- | --- | --- | --- | --- |
| 10-HDHA | 23.83±9.75 | 11.24±6.13 | 19.63±10.28 | 20.69 | <0.001 | 1.212 |
| 11(12)-DiHET | 1.63±0.80 | 3.03±1.88 | 2.37±1.55 | 8.92 | <0.001 | 0.837 |
| 11(12)-DiHETE | 0.18±0.08 | 0.16±0.09 | 0.15 ±0.06 | 1.738 | 0.180 | 0.734 |
| 11(S)-HETE | 31.60±19.15 | 14.72±8.92 | 20.47±8.59 | 16.98 | <0.001 | 1.232 |
| 11,12-EET | 32.21±14.63 | 68.96±32.24 | 35.48±17.12 | 32.11 | <0.001 | 1.408 |
| 11-HDHA | 19.46±10.72 | 7.37±2.88 | 13.25±6.90 | 25.71 | <0.001 | 1.266 |
| 11-HEDE | 2.21±0.98 | 1.48±0.49 | 2.00±0.56 | 10.94 | <0.001 | 0.969 |
| 11-HEPE | 2.64±1.45 | 1.63±0.97 | 2.16±1.03 | 7.46 | <0.001 | 1.001 |
| 11β-PGF2α | 0.55±0.23 | 0.58±0.28 | 0.54±0.23 | 0.243 | 0.784 | 0.302 |
| 12(13)-DiHOME | 14.92±8.59 | 26.40±17.67 | 20.17±12.20 | 7.415 | <0.001 | 0.896 |
| 12,13-EpOME | 158.03±71.45 | 292.35±151.67 | 258.62±149.84 | 11.59 | <0.001 | 0.970 |
| 12-HEPE | 2.69±1.79 | 2.59±1.52 | 3.62±2.23 | 3.692 | 0.028 | 1.085 |
| 12-HETE | 53.43±26.14 | 56.00±26.26 | 94.91±51.68 | 16.03 | <0.001 | 1.373 |
| 13(14)-DiHDPE(A) | 0.31±0.18 | 0.46±0.31 | 0.37±0.27 | 3.283 | 0.041 | 0.754 |
| 13(S)-HODE | 121.26±75.95 | 104.04±55.38 | 132.12±61.95 | 1.900 | 0.154 | 0.782 |
| 13(S)-HpODE | 27.17±12.66 | 20.55±10.74 | 4.82±2.61 | 55.98 | <0.001 | 1.644 |
| 13-HDHA | 22.20±13.26 | 7.93±3.81 | 13.62±6.12 | 27.16 | <0.001 | 1.342 |
| 13-HOTrE | 25.39±18.69 | 60.57±45.48 | 38.78±18.78 | 13.66 | <0.001 | 1.136 |
| 13-oxo-ODE | 0.36±0.24 | 0.77±0.41 | 0.14±0.09 | 52.08 | <0.001 | 1.643 |
| 14(15)-DiHET | 3.70±2.10 | 4.77±2.89 | 3.27±2.27 | 4.02 | 0.021 | 0.922 |
| 14(15)-DiHETE | 0.12±0.08 | 0.12±0.07 | 0.10±0.06 | 1.480 | 0.232 | 0.693 |
| 14(15)-EpETE | 2.68±1.35 | 6.08±3.84 | 5.18±3.61 | 12.65 | <0.001 | 1.053 |
| 14(S)-HDHA | 45.92±24.39 | 52.85±44.39 | 138.31±82.79 | 33.73 | <0.001 | 1.590 |
| 14,15-EET | 52.32±25.71 | 117.19±67.37 | 66.51±48.26 | 18.53 | <0.001 | 1.311 |
| 15(S)-HETrE | 6.81±2.79 | 4.04±1.91 | 5.94±2.37 | 14.14 | <0.001 | 1.206 |
| 15-HEDE | 2.41±1.22 | 1.39±0.37 | 1.98±0.56 | 16.12 | <0.001 | 1.128 |
| 15-HEPE | 1.63±1.07 | 0.91±0.51 | 1.27±0.69 | 8.224 | <0.001 | 1.087 |
| 15-HETE | 45.67±19.94 | 26.59±14.24 | 34.53±11.77 | 14.92 | <0.001 | 1.149 |
| 15-keto-PGF1α | 4.32±1.74 | 3.48±1.74 | 3.19±1.24 | 5.485 | 0.005 | 0.790 |
| 15-oxo-ETE | 3.67±1.86 | 2.28±1.31 | 2.20±1.25 | 12.15 | <0.001 | 0.770 |
| 16(17)-EpDPE | 13.32±7.95 | 24.91±18.01 | 22.05±16.85 | 6.512 | 0.002 | 0.865 |
| 16-HDHA | 23.04±14.03 | 8.29±3.80 | 13.96±6.79 | 25.80 | <0.001 | 1.321 |
| 16-HETE | 1.59±0.96 | 1.59±0.99 | 1.52±0.90 | 0.068 | 0.934 | 0.695 |
| 17(18)-DiHETE | 0.27±0.18 | 0.46±0.29 | 0.44±0.22 | 7.670 | <0.001 | 0.716 |
| 17(18)-EpETE | 1.77±1.04 | 3.20±2.00 | 3.04±2.38 | 6.873 | 0.002 | 0.974 |
| 17-HDHA | 65.67±32.96 | 30.85±16.63 | 52.75±27.74 | 17.43 | <0.001 | 1.210 |
| 17-HETE | 0.48±0.33 | 0.45±0.34 | 0.44±0.32 | 0.155 | 0.856 | 0.581 |
| 18-HEPE | 3.42 ±1.82 | 2.30±1.01 | 2.99±1.40 | 6.068 | 0.003 | 0.958 |
| 18-HETE | 0.84±0.52 | 0.86±0.44 | 0.70±0.42 | 1.453 | 0.238 | 0.771 |
| 19(20)-DiHDPE(A) | 1.75±1.02 | 2.15±1.29 | 2.03±1.31 | 1.118 | 0.330 | 0.471 |
| 19(20)-EpDPE(A) | 9.45±9.36 | 14.80±10.62 | 11.62±7.89 | 3.302 | 0.040 | 0.757 |
| 19(R)-hydroxyPGF2α | 10.61±4.86 | 11.12±6.51 | 10.78±3.68 | 0.103 | 0.903 | 0.451 |
| 2,3-dinor-8-iso-PGF2α | 14.03±5.78 | 12.19±4.57 | 11.73±4.06 | 2.519 | 0.085 | 0.584 |
| 20-COOH-ARA | 32.67±20.64 | 26.02±18.62 | 28.01±17.20 | 1.308 | 0.274 | 0.471 |
| 20-HDHA | 40.81±230.2 | 21.26±14.23 | 35.10±19.95 | 10.72 | <0.001 | 0.757 |
| 20-hydroxyPGF2α | 6.51±2.99 | 5.66±1.35 | 6.14±2.08 | 1.431 | 0.243 | 0.471 |
| 4-HDHA | 39.91±26.73 | 15.57±11.77 | 23.62±9.56 | 19.53 | <0.001 | 1.203 |
| 5,6-DIHETE | 2.06±1.54 | 3.10±1.80 | 3.04±1.54 | 4.914 | 0.009 | 0.659 |
| 5,6-DiHETrE | 1.08±0.64 | 1.07±0.66 | 1.15±0.69 | 0.176 | 0.839 | 0.309 |
| 5-HEPE | 3.84±2.47 | 1.77±0.98 | 2.20±1.51 | 15.19 | <0.001 | 1.022 |
| 5-HETE | 29.88±10.10 | 38.63±10.60 | 64.90±25.80 | 45.32 | <0.001 | 1.552 |
| 5-HETrE | 0.81±0.53 | 0.41±0.23 | 0.53±0.28 | 10.69 | <0.001 | 0.867 |
| 5-oxo-ETE | 1.38±0.29 | 5.13±1.87 | 8.12±2.40 | 146.3 | <0.001 | 1.704 |
| 6keto-PGF1α | 12.53±10.46 | 6.29±2.30 | 4.99±1.26 | 16.53 | <0.001 | 1.089 |
| 7-HDHA | 17.77±9.86 | 7.71±4.40 | 11.41±6.05 | 20.22 | <0.001 | 1.242 |
| 8(9)-DiHET | 1.68±0.98 | 1.88±1.14 | 1.72±0.97 | 0.428 | 0.653 | 0.405 |
| 8(S)-HETrE | 5.07±1.95 | 2.90±1.08 | 4.28±1.95 | 15.25 | <0.001 | 1.130 |
| 8,9-EET | 15.65±11.68 | 41.10±28.03 | 20.44±14.67 | 18.05 | <0.001 | 1.165 |
| 8-HDHA | 40.66±22.50 | 15.50±8.17 | 27.88±16.17 | 22.33 | <0.001 | 1.191 |
| 8-HEPE | 1.32±0.83 | 0.75±0.48 | 0.90±0.51 | 8.087 | <0.001 | 0.965 |
| 8-HETE | 31.87±18.58 | 13.64±5.11 | 20.98±8.25 | 22.45 | <0.001 | 1.244 |
| 9(S),10(S),13(S)-TriHOME | 7.59±4.98 | 6.23±3.91 | 5.44±3.80 | 2.596 | 0.079 | 0.396 |
| 9(S),12(S),13(S)-TriHOME | 16.15±10.95 | 15.21±10.95 | 11.71±8.81 | 1.995 | 0.141 | 0.396 |
| 9,10-DiHOME | 53.34±37.61 | 76.43±38.59 | 57.01±28.94 | 4.938 | 0.009 | 0.835 |
| 9-HEPE | 1.55±0.97 | 0.94±0.55 | 1.23±0.71 | 6.176 | 0.003 | 1.048 |
| 9-HETE | 28.65±22.15 | 12.35±6.24 | 15.76±7.77 | 14.82 | <0.001 | 1.099 |
| 9-HODE | 141.90±115.92 | 103.80±39.80 | 133.53±83.66 | 2.184 | 0.117 | 0.656 |
| 9-HOTrE | 7.07±6.28 | 8.56±6.02 | 5.82±2.70 | 2.629 | 0.077 | 0.801 |
| 9-oxo-OTrE | 1.43±0.74 | 2.21±1.44 | 2.72±1.99 | 7.458 | <0.001 | 0.737 |
| ARA | 64771.0±25439.5 | 80845.0±16985.9 | 107884.0±26882.8 | 34.35 | <0.001 | 1.446 |
| DHA | 50151.9±12892.8 | 47240.8±15264.0 | 48462.4±18538.6 | 0.345 | 0.709 | 0.364 |
| D-γ-LA | 25808.9±8470.2 | 27218.7±10607.3 | 31582.4±12308.3 | 3.197 | 0.045 | 0.887 |
| EPA | 4781.4±2498.3 | 5125.7±3221.9 | 5025.1±3138.3 | 0.142 | 0.868 | 0.471 |
| tetranor-12(S)-HETE | 0.41±0.26 | 0.36±0.28 | 0.42±0.21 | 0.693 | 0.502 | 0.557 |
| tetranor-PGFM | 33.13±17.95 | 23.81±10.82 | 23.53±8.36 | 6.467 | 0.002 | 0.917 |
| trans-EKODE-(E)-Ib | 18.40±9.69 | 22.32±14.39 | 20.56±9.96 | 1.115 | 0.332 | 0.379 |
| γ-LA | 1777.8±1220.4 | 2209.3±1142.0 | 3456.1±2137.9 | 12.17 | <0.001 | 0.932 |

**Supplemental Table S7.** Plasma levels of the detected oxylipins in the patients of validation cohort.

| Oxylipins (nM) | CON  (n=20) | T2DM  (n=20) | DKD  (n=20) | F | *P* | VIP  value |
| --- | --- | --- | --- | --- | --- | --- |
| 10-HDHA | 24.54±7.71 | 15.33±5.75 | 18.96±8.44 | 3.278 | 0.045 | 1.136 |
| 11(12)-DiHET | 0.52±0.11 | 0.69±0.30 | 0.68±0.30 | 4.522 | 0.015 | 1.024 |
| 11(S)-HETE | 41.89±16.20 | 36.13±10.03 | 75.50±42.57 | 9.668 | <0.001 | 1.204 |
| 11-HDHA | 18.42±5.27 | 11.46±3.51 | 14.83±7.48 | 2.305 | 0.109 | 1.181 |
| 11-HEDE | 2.12±0.56 | 2.48±1.03 | 3.80±1.05 | 15.11 | <0.001 | 1.212 |
| 11-HEPE | 7.12±2.18 | 5.38±1.85 | 6.36±2.09 | 0.827 | 0.443 | 0.559 |
| 12(13)-DiHOME | 10.72±4.66 | 9.13±5.04 | 9.33±3.50 | 0.696 | 0.503 | 0.458 |
| 12-HEPE | 4.65±2.77 | 3.78±1.99 | 8.03±4.98 | 5.697 | 0.006 | 1.064 |
| 12-HETE | 80.39±32.77 | 82.96±38.29 | 159.87±88.67 | 12.62 | <0.001 | 1.204 |
| 13(14)-DiHDPE(A) | 0.10±0.02 | 0.11±0.08 | 0.14±0.08 | 5.88 | 0.005 | 0.756 |
| 13(S)-HODE | 638.89±216.13 | 530.99±317.12 | 470.16±256.33 | 3.578 | 0.034 | 0.593 |
| 13(S)-HOTrE(γ) | 27.15±9.66 | 25.44±6.11 | 32.74±9.42 | 2.271 | 0.112 | 0.699 |
| 13-HDHA | 27.80±8.95 | 17.16±5.94 | 23.42±12.21 | 1.237 | 0.298 | 1.136 |
| 13-HOTrE | 218.46±85.91 | 138.15±97.17 | 115.01±69.47 | 10.93 | <0.001 | 0.778 |
| 14(15)-DiHET | 0.57±0.13 | 0.79±0.51 | 0.85±0.39 | 8.797 | <0.001 | 0.949 |
| 14(S)-HDHA | 38.36±11.28 | 32.60±19.21 | 35.52±18.14 | 0.277 | 0.759 | 0.849 |
| 15(S)-HETrE | 8.37±2.91 | 7.59±1.59 | 11.17±4.73 | 3.979 | 0.024 | 1.142 |
| 15-HEPE | 3.12±1.91 | 2.43±1.07 | 4.91±2.91 | 4.071 | 0.022 | 1.127 |
| 15-HETE | 51.40±18.96 | 48.92±12.37 | 86.48±45.25 | 8.897 | <0.001 | 1.166 |
| 16-HDHA | 27.24±8.47 | 17.42±5.96 | 21.93±11.81 | 1.996 | 0.145 | 1.115 |
| 16-HETE | 3.54±0.70 | 3.12±1.12 | 4.61±1.09 | 10.61 | <0.001 | 1.022 |
| 17(18)-DiHETE | 0.10±0.05 | 0.16±0.10 | 0.09±0.06 | 0.558 | 0.576 | 0.811 |
| 17-HDHA | 68.57±22.76 | 42.60±13.85 | 59.61±32.65 | 0.765 | 0.470 | 1.129 |
| 17-HETE | 1.06±0.26 | 1.08±0.48 | 1.63±0.44 | 20.00 | <0.001 | 1.072 |
| 18-HEPE | 4.95±1.92 | 3.60±1.08 | 6.21±3.07 | 1.903 | 0.158 | 0.989 |
| 18-HETE | 1.61±0.44 | 1.40±0.64 | 2.06±0.62 | 5.282 | 0.008 | 0.884 |
| 19(20)-DiHDPE(A) | 0.90±0.26 | 0.90±0.41 | 0.70±0.29 | 3.591 | 0.034 | 0.770 |
| 19(20)-EpDPE(A) | 1.52±0.68 | 1.07±0.46 | 1.38±0.72 | 0.255 | 0.776 | 0.852 |
| 19(R)-hydroxyPGF2α | 4.53±0.97 | 4.35±1.35 | 7.72±2.91 | 19.64 | <0.001 | 1.255 |
| 20-COOH-ARA | 64.15±29.46 | 46.08±29.59 | 42.17±23.28 | 4.245 | 0.019 | 0.866 |
| 20-HDHA | 34.41±10.57 | 23.74±11.11 | 23.73±12.89 | 5.854 | 0.005 | 1.021 |
| 20-hydroxyPGF2α | 4.22±1.20 | 4.67±1.47 | 11.06±5.71 | 26.33 | <0.001 | 1.256 |
| 4-HDHA | 92.13±32.75 | 43.37±14.33 | 56.31±29.67 | 8.484 | <0.001 | 1.251 |
| 5,6-DIHETE | 0.46±0.24 | 0.65±0.47 | 0.80±0.40 | 8.327 | <0.001 | 0.840 |
| 5,6-DiHETrE | 0.31±0.09 | 0.40±0.18 | 0.33±0.14 | 16.48 | <0.001 | 1.150 |
| 5-HEPE | 4.04±1.87 | 3.01±1.16 | 4.68±2.85 | 0.546 | 0.582 | 0.886 |
| 5-HETE | 38.03±11.66 | 45.74±11.61 | 92.41±25.67 | 55.88 | <0.001 | 1.409 |
| 5-oxo-ETE | 2.01±0.46 | 6.06±1.77 | 8.23±2.73 | 55.67 | <0.001 | 1.303 |
| 6keto-PGF1α | 27.28±8.91 | 40.94±19.09 | 32.95±12.87 | 1.978 | 0.148 | 0.986 |
| 7-HDHA | 18.09±5.45 | 10.92±3.72 | 14.41±7.15 | 2.451 | 0.095 | 1.214 |
| 8(9)-DiHET | 0.79±0.21 | 0.90±0.37 | 0.84±0.30 | 0.311 | 0.340 | 0.830 |
| 8(S)-HETrE | 4.68±1.79 | 4.15±1.34 | 4.87±2.58 | 0.055 | 0.946 | 0.660 |
| 8,9-EET | 3.24±0.93 | 12.86±6.99 | 19.29±6.26 | 125.8 | <0.001 | 1.351 |
| 8-HDHA | 37.63±11.69 | 22.59±7.77 | 28.75±14.59 | 3.239 | 0.047 | 1.204 |
| 8-HEPE | 2.52±0.66 | 1.91±0.98 | 3.51±2.02 | 3.963 | 0.025 | 1.056 |
| 8-HETE | 38.45±15.30 | 32.18±11.92 | 70.06±39.24 | 9.950 | <0.001 | 1.286 |
| 9(S),10(S),13(S)-TriHOME | 2.74±0.92 | 2.55±1.02 | 2.23±0.98 | 1.981 | 0.147 | 0.526 |
| 9(S),12(S),13(S)-TriHOME | 7.48±2.67 | 5.28±1.65 | 4.52±1.60 | 10.48 | <0.001 | 1.107 |
| 9,10-DiHOME | 109.20±46.91 | 81.90±31.74 | 51.53±21.03 | 13.73 | <0.001 | 1.067 |
| 9-HEPE | 2.78±1.35 | 2.09±0.97 | 4.04±2.79 | 2.798 | 0.069 | 0.526 |
| 9-HETE | 21.27±7.07 | 17.98±6.63 | 36.71±19.98 | 9.552 | <0.001 | 1.274 |
| 9-HODE | 499.09±162.44 | 429.43±241.90 | 358.13±178.60 | 4.693 | 0.013 | 0.526 |
| 9-HOTrE | 26.25±11.61 | 16.33±9.71 | 11.75±5.00 | 14.28 | <0.001 | 1.107 |
| 9-oxo-OTrE | 24.77±9.71 | 18.82±6.99 | 16.13±9.42 | 5.382 | 0.007 | 0.526 |
| ARA | 63803.8±11036.0 | 76657.0±14531.2 | 96984.9±27906.6 | 32.87 | <0.001 | 1.212 |
| DHA | 44347.6±5989.5 | 35515.7±6545.7 | 34357.0±9586.1 | 12.20 | <0.001 | 0.953 |
| D-γ-LA | 20146.2±5888.4 | 19039.8±4.56.2 | 21423.2±4406.5 | 0.367 | 0.694 | 0.543 |
| EPA | 5130.3±2499.5 | 4355.5±1618.9 | 4440.0±2613.8 | 0.493 | 0.613 | 0.529 |
| LA | 84292.0±17027.0 | 72742.9±11506.4 | 73541.7±19952.2 | 2.364 | 0.103 | 0.821 |
| PGA2 | 1.17±0.56 | 1.48±0.47 | 2.01±0.91 | 9.662 | <0.001 | 1.048 |
| RvE1 | 51.95±7.01 | 48.59±11.86 | 57.12±14.50 | 1.727 | 0.187 | 0.625 |
| tetranor-12(S)-HETE | 0.87±0.23 | 0.70±0.34 | 0.86±0.27 | 0.009 | 0.991 | 0.547 |
| tetranor-PGFM | 26.38±6.06 | 22.55±6.26 | 33.04±9.52 | 5.414 | 0.007 | 1.181 |
| α-LA | 43024.9±13072.8 | 27652.0±11806.5 | 29043.3±10882.8 | 8.495 | <0.001 | 0.967 |
| γ-LA | 3333.9±1755.1 | 2480.6±1045.0 | 2171.9±649.4 | 4.102 | 0.022 | 0.676 |
